# Clinical implications of RAB13 expression in pan-cancer based on multi-databases integrative analysis

**DOI:** 10.1038/s41598-023-43699-2

**Published:** 2023-10-06

**Authors:** Xu-dong Zhang, Zhong-yuan Liu, Kai Luo, Xiang-kun Wang, Mao-sen Wang, Shuai Huang, Ren-feng Li

**Affiliations:** Departments of Hepatobiliary and Pancreatic Surgery, The First Affiliated Hospital of Zhengzhou University, Zhengzhou, 450052 Henan Province China

**Keywords:** Oncogenes, Tumour biomarkers, Cancer

## Abstract

Worldwide, cancer is a huge burden, and each year sees an increase in its incidence. RAB (Ras-related in brain) 13 is crucial for a number of tumor types. But more research on RAB13's tumor-related mechanism is still required. This study's goal was to investigate RAB13's function in human pan-cancer, and we have also preliminarily explored the relevant mechanisms. To investigate the differential expression, survival prognosis, immunological checkpoints, and pathological stage of RAB13 in human pan-cancer, respectively, databases of TIMER2.0, GEPIA 2, and UALCAN were employed. CBioPortal database was used to analyze the mutation level, meanwhile, PPI network was constructed based on STRING website. The putative functions of RAB13 in immunological infiltration were investigated using single sample gene set enrichment analysis (ssGSEA). The mechanism of RAB13 in hepatocellular cancer was also briefly investigated by us using gene set enrichment analysis (GSEA). RAB13 was differentially expressed in a number of different cancers, including liver hepatocellular carcinoma (LIHC), stomach adenocarcinoma (STAD), etc. Additionally, RAB13 overexpression in LGG and LIHC is associated with a worse prognosis, including overall survival (OS) and disease-free survival (DFS). Then, we observed that early in BLCA, BRAC, CHOL, ESCA, HNSC, KICH, KIRC, LIHC, LUAD, LUSC, and STAD, the level of RAB13 expression was raised. Next, we found that “amplification” was the most common mutation in *RAB13*. The expression of SLC39A1, JTB, SSR2, SNAPIN, and RHOC was strongly positively linked with RAB13, according to a correlation study. RAB13 favorably regulated B cell, CD8 + T cell, CD4 + T cell, macrophage, neutrophil, and dendritic cell in LIHC, according to immune infiltration analysis. Immune checkpoint study revealed a positive correlation between RAB13 expression and PD1, PDL1, and CTLA4 in LIHC. According to GSEA, RAB13 is involved in a number of processes in LIHC, including MTORC1 signaling, MYC targets v1, G2M checkpoint, MITOTIC spindle, DNA repair, P53 pathway, glycolysis, PI3K-AKT-MTOR signaling, etc. RAB13 is a possible therapeutic target in LIHC and can be used as a prognostic marker.

## Introduction

Using computational tools to examine and interpret biological data, such as DNA, RNA, and protein sequences, is bioinformatics analysis. This requires a variety of approaches, such as data mining, pattern recognition, machine learning, statistical analysis, and visualisation. Bioinformation analysis brings great convenience to clinical and scientific research^1^. Firstly, it helps to understand complex biological systems and processes involving various biological functions; Secondly, it can identify potential drug targets; It can also identify biomarkers and predict disease progression to aid in the diagnosis and prognosis of diseases; Finally, a specific treatment plan is supplied based on the patient's genetic traits in order to achieve optimal patient care.

Cancer remains a worldwide problem, which brings great pain to the patients and a great economic burden to society. According to recent studies, there were 19.3 million new cancer cases and nearly 10 million cancer deaths in 2020, and the global cancer burden is expected to reach 28.4 million cases by 2040^2,3^. There are many types of tumors, and the mechanism of tumor occurrence and development is very complex. This poses a great challenge for the treatment of tumors. Researches on the molecular mechanisms of tumorigenesis still have a long way to go. Therefore, it is necessary to strengthen the research of tumor. It has been reported that Ras-related in brain (*RAB*) proteins family is the primary regulator of vesicle trafficking^4^. Vesicle transport plays an important role in tumor invasion, migration, metabolism, autophagy, exosome secretion and drug resistance^5,6^. RAB13, a member of the RAB family, has recently attracted attention. Studies have shown that *RAB13* can enhance glucose utilization in multiple myeloma^7,8^. Small extracellular vesicles (sEVs) have been shown to promote cell–cell communication, which has crucial implications for the interactions between the tumor microenvironment and tumor growth and metastasis. James G. Patton et al.^9^ demonstrated that by controlling the release of sEVs, RAB13 aids in the proliferation and invasion of colorectal cancer cells. A study by Johanna Ivaska et al.^10^ found that active β1 integrin can mediate cell–matrix interaction, and RAB13 enhances its circulation to the plasma membrane to promote breast cancer invasion. Its transport is crucial for the dynamic regulation of cell adhesion, migration, and malignant processes. In addition, by influencing the tumor microenvironment, RAB13 maintains breast cancer stem cells^11^. Another study showed that interruption of the RAB13-DENND2B transport pathway significantly reduces the migration of highly invasive breast cancer cells in vivo^12^. Furthermore, *RAB13* was reported to be the chemotherapeutic resistance in gastric cancer cells^13^. These studies amply supported the crucial part RAB13 plays in malignancies. Although RAB13's function and mechanism in a few cancers are well understood, further research is still needed to determine how RAB13 works in pan-cancer.

In this research, we carried out a thorough data-mining analysis based on examination of the expression profile of RAB13. Then, we investigated the correlation between *RAB13* and survival status, pathological stage, genetic mutations, and immune infiltration. Finally, we explored the mechanisms and signaling pathways involved. Overall, our research has offered a complete examination of RAB13 and fresh information on its function in malignancies.

## Materials and methods

### RAB13 expression patterns in different types of cancers

TIMER2.0 (Tumor Immune Estimation Resource, version 2, http://timer.cistrome.org/)^14^ database was used to examine distinct expression of *RAB13* mRNA in pan-cancer and normal tissue specimens. To determine the difference between the expression of the cancer and paracancer genes, the Wilcoxon test was performed. GEPIA2 (Gene Expression Profiling Interactive Analysis, version 2, http://gepia2.cancer-pku.cn/#index)^15^ database was used to compensate for the limitations of TIMER2.0. log2FC (fold change) cutoff Set to 1 and p-value Cutoff Set to 0.01. We utilized UALCAN database (The University of ALabama at Birmingham CANcer data analysis Portal, http://ualcan.path.uab.edu/analysis-prot.html)^16^ to examine distinct expression of RAB13 in terms of proteins. Standard deviations from the median for samples for a given cancer type are shown by z-values. After being standardised within each sample profile, the log2 spectral count ratio values from CPTAC were then normalised across samples.

### Survival prognosis analysis

We did survival analyses with GEPIA2 database, including OS and DFS. Cutoff-high and cutoff-low values were set to 75% and 25%, respectively. And we calculated the hazard ratio according to the Cox PH model. As a result, the cohort can be split into groups with high and low expression. In order to quantify differences in OS and DFS across a range of tumor types, the log-rank test was performed.

### Pathological stage and RAB13 correlation analysis

The correlation between RAB13 and the pathological stage of the tumor was done using the UALCAN program. We entered RAB13 into the UALCAN online label and examined the association between the various cancer types provided from the TCGA experiment.

### Genetic alterations analysis

We used the cBioPortal (https://www.cbioportal.org/)^17^ tool, a sophisticated dynamic content resource for cancer omics predictive analytics, to undertake genetic alteration analysis. This platform offers researchers a clear remote console and humanistic visual interaction. Information about the RAB13 mutation, including the alteration frequency, mutation type, copy number alteration, altered site, and 3D (three dimensional) structure, was collected from this website. In addition, we compared OS with RAB13 gene alterations and without RAB13 gene alterations in the survival module.

### Analysis of immune infiltration and infiltration checkpoints

By using single sample gene set enrichment analysis, the levels of immune cell and immune regulatory factor infiltration were assessed. The R Bioconductor package Gene Set Variation Analysis (GSV A, v.3.5)^18^ was used to carry out the ssGESA. After that, the link between RAB13 and B cell, CD8 + T cell, CD4 + T cell, macrophage, neutrophil, and dendritic cell was confirmed using the TIMER (https://cistrome.shinyapps.io/timer/) database. Immune checkpoints analysis was also carried out by TIMER database.

### Study of the gene enrichment in RAB13

We created a protein–protein interaction (PPI) network using the STRING (https://cn.string-db.org/)^19^ website. In the Protein Name module, we typed RAB13, and in the Organisms module, we selected Homo sapiens. The advanced settings are shown below: Network Type: full STEING network; Required score: medium confidence (0.400); Size cutoff: no more than 20 interactors.

We downloaded the top 100 *RAB13*-correlated genes from GEPIA2 database. Then we analysed the correlation between part of the genes and *RAB13* by scatter plot analysis. Next, we screened and sorted out the downloaded data. Finally, we used Xiantao academic (https://www.xiantao.love/products) to draw the heatmap of the expression profile of screening genes and carried out GO/KEGG pathway analysis.

### GSEA

GSEA^20^ was carried out using the procedures outlined by Dadousis et al.^21^. 54,615 features make up the dataset (genes). All 54,615 characteristics were used because no probe set =  > gene symbol collapsing was requested. Gene set size filters (min = 15, max = 500) resulted in filtering out 0/50 gene sets. The ReactomePA Package^22^ was used to process the data. The correction for the false discovery rate (< 0.25) was regarded as statistically significant.

### Statistical analysis

In order to do statistical analysis, we used IBM SPSS Statistics 21 and R v4.2.1 R v4.2.1 (https://www.r-project.org/). For the survival analysis, the log-rank test was employed. Immunological checkpoint analysis and immune infiltration analysis both employed spearman correlation coefficients. Statistics were judged significant at *P* < 0.05.

## Results

### Expression of *RAB13* in human pan-cancer

**Figure 1:**
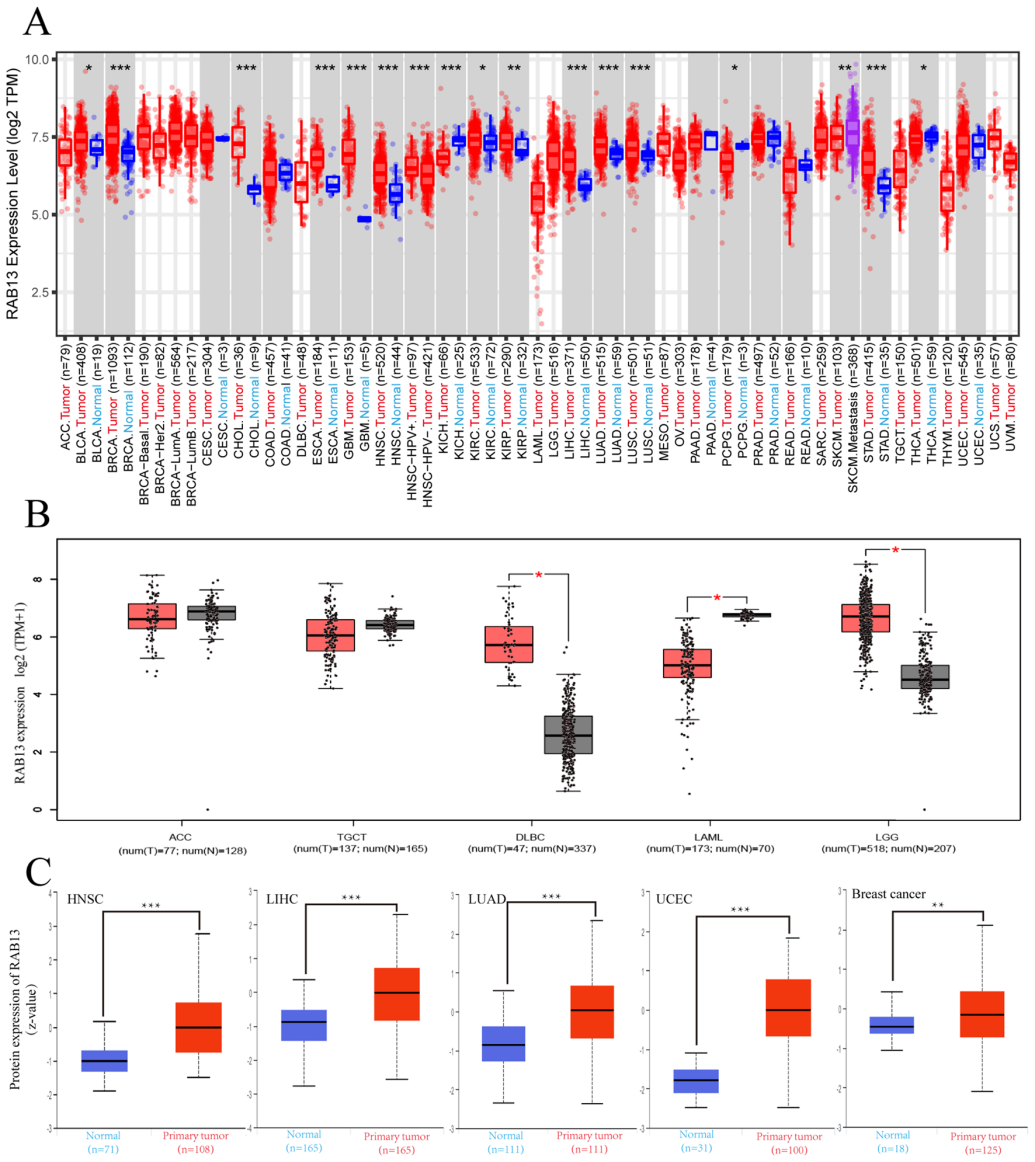
RAB13 protein and RNA levels in various cancer types. (**A**) The TIMER database's analysis of RAB13 transcription levels in various cancer types. (**B**) TIMER database supplement based on GEPIA 2 database. (**C**) The CPTAC database's protein level of RAB13 in various cancer types. *P < 0.05 **P < 0.01 ***P < 0.001.

The differential expression of RAB13 between tumor and surrounding normal tissues was investigated across all TCGA cancers using the TIMER2.0 database. The expression of RAB13 in tumor tissues was higher than that in corresponding para-cancerous tissues in BLCA, BRCA, CHOL, ESCA, GBM, HNSC, KIRC, KIRP, LIHC, LUAD, LUSC, and STAD (all *P* < 0.05, Fig. 1A). However, compared to their surrounding normal tissues, KICH, PCPG, and THCA had reduced expression levels of RAB13 (all *P* < 0.05, Fig. 1A). GTEx dataset was used as replenishment due to some cancers lacking of corresponding normal samples in the TIMER database. As illustrated in Fig. 1B, its expression in DLBC, LAML and LGG had differences (all *P* < 0.05).

Furthermore, according to the CPTAC, we investigated the RAB13 protein's differential expression in tumor and normal tissues. The findings demonstrated that, in comparison to normal tissues, the expression of RAB13's total protein was considerably higher in tumor tissues from the HNSC, LIHC, LUAD, UCEC and breast cancer (all *P* < 0.05, Fig. 1C).

### RAB13's prognostic significance for all malignancies

**Figure 2:**
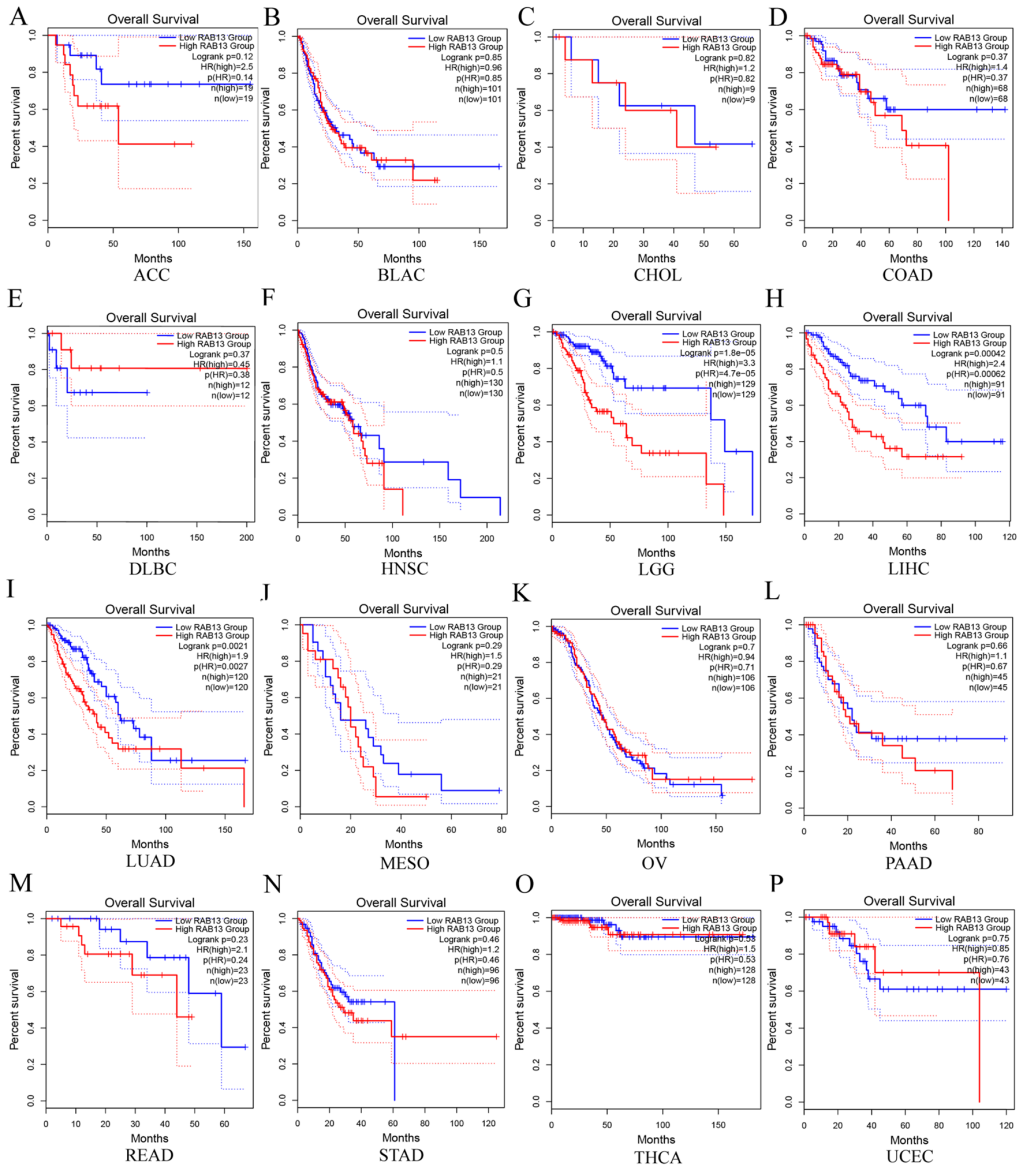
Based on data from the GEPIA 2 database, the relationship between RAB13 expression and OS in various malignancies. (**A**) ACC, (**B**) BLAC, (**C**) CHOL, (**D**) COAD, (**E**) DLBC, (**F**)HNSC, (**G**)LGG, (**H**) LIHC, (**I**) LUAD, (**J**) MESO, (**K**) OV, (**L**) PAAD, (**M**) READ, (**N**) STAD, (**O**) THCA, (**P**) UCEC.

We used GEPIA 2 to analyze the survival of RAB13 in pan-cancer to look into the relationship between RAB13 expression and prognostic value. According to Fig. 2, elevated *RAB13* expression predicted worse OS in LGG, LIHC and LUAD (all *P* < 0.001). The increased expression of RAB13 predicted a worse prognosis for DFS in ACC, LGG, LIHC and READ (all *P* < 0.05, Fig. S1). The aforementioned information indicated that *RAB13* plays a malignant character in LGG and LIHC, consequently, it might be an effective therapeutic candidate for cancer.

### RAB13 expression varies depending on the stage of the cancer

**Figure 3:**
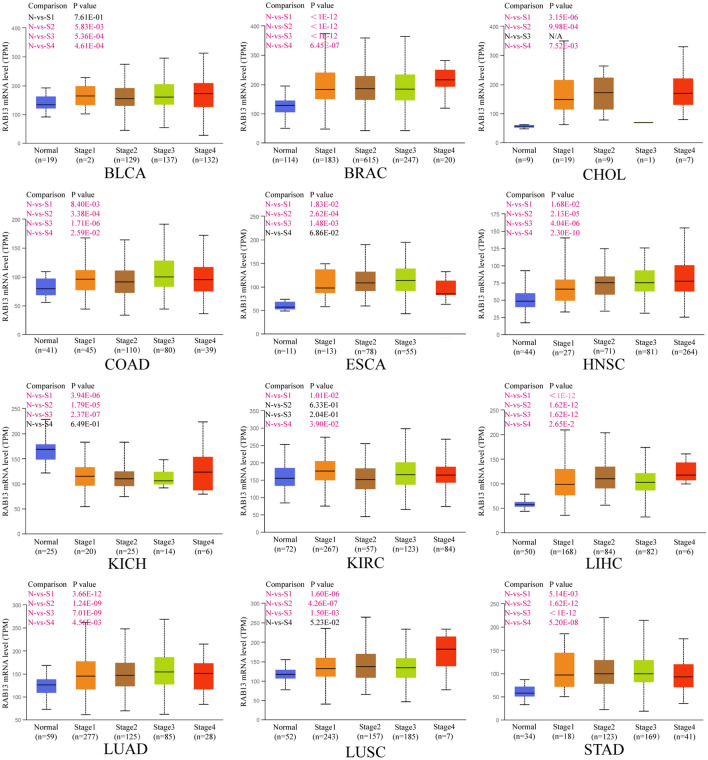
UALCAN database-based correlation between RAB13 expression and pathologic stages in various malignancies. *N* normal, *S* stage.

Then, we used UALVAN database to retrieve the data of TCGA cancers to investigate the relationship between RAB13 expression and the pathological stage of various malignancies. RAB13 was discovered to be considerably more expressed in BRCA, CHOL, COAD, ESCA, HNSC, KIRC, LIHC, LUAD, LUSC, and STAD than in normal tissue (all *P* < 0.05, Fig. 3). However, there was no discernible difference between the early and late stages. What is noteworthy was that the expression of *RAB13* were lower than normal tissue in KICH (*P* < 0.001; Fig. 3). According to the aforementioned findings, RAB13 may promote the development of different cancer types.

### Genetic mutations analysis of* RAB13*

**Figure 4:**
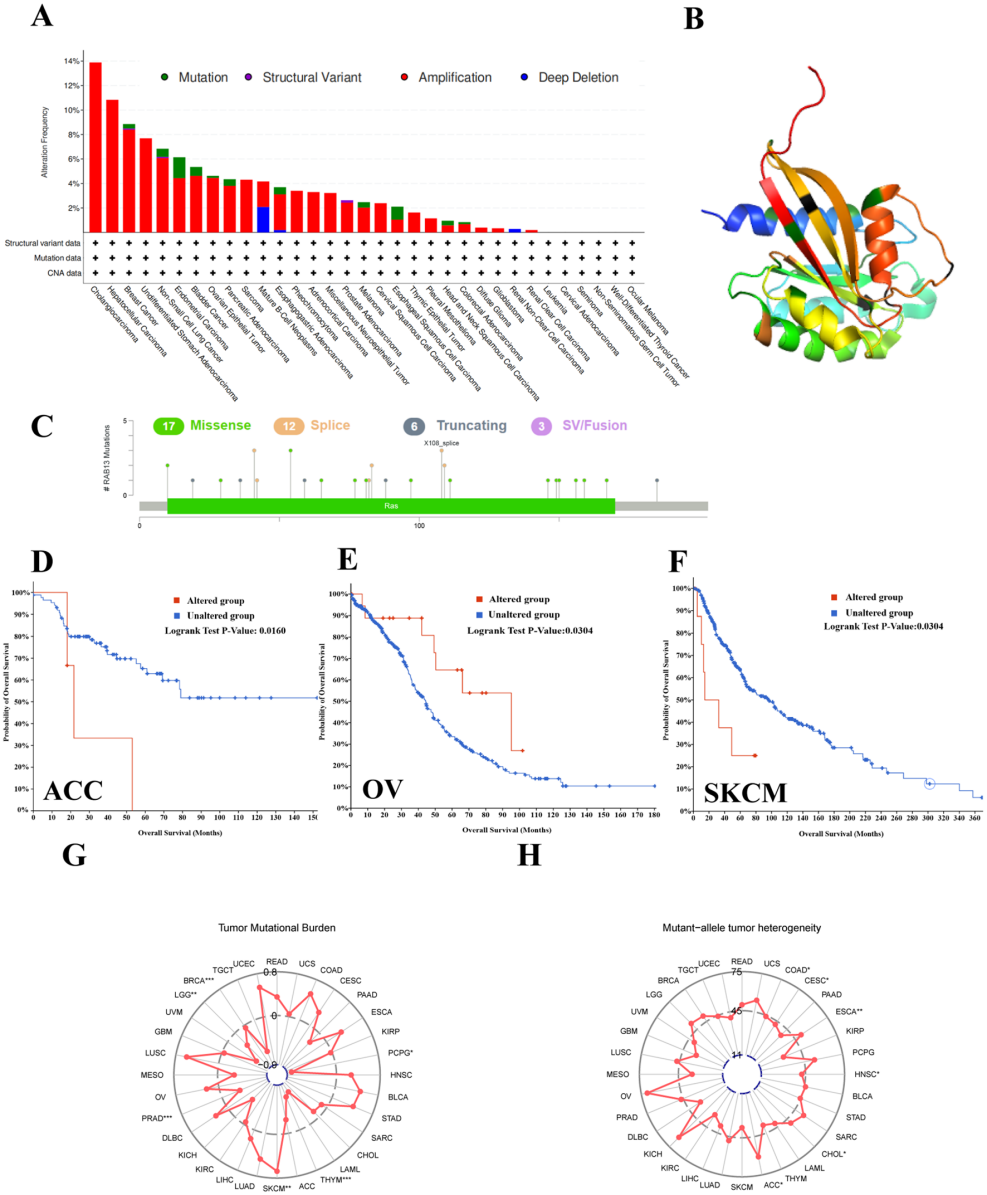
Mutations in cBioPortal's RAB13 database, which are found in a variety of malignancies. (**A**) frequency of change as a function of mutation type. (**B**) Sites of RAB13 genetic mutation. (**C**) 3D model of the mutated location. (**D**) RAB13 gene mutation and ACC's OS. (**E**) RAB13 gene mutation and OV's OS. (**F**) RAB13 gene mutation and SKCM's OS. (**G**) A radar map showing how the amounts of mutations in a tumor relates to the amount of RAB13 expression. (**H**) Expression of RAB13 as a radar for detecting tumors with a mutant allele.

It is common knowledge that transgenes have a significant impact on the formation and growth of cancers. Therefore, illustrating the mutation features and biological functions of *RAB13* are of profound significance for our deeper understanding of tumors. We conducted a thorough investigation of the status of RAB13 gene changes in human pan-cancer using the cBioPortal database. We found that one of the most significant single determinants for change in many malignancies was the amplification of RAB13. With a frequency of > 10%, CHOL and LIHC had the most instances of the "amplification" kind of CAN among them (Fig. 4A). Then, the form and location of *RAB13* genetic mutations were further investigated. The results are shown in Fig. 4C. 17 cases of missense and 12 cases of splice were found, which were the main type of genetic alteration of *RAB13*. Figure 4B depicts the RAB13 3D structure. Finally, we investigated whether or not gene changes were associated with OS. The ACC and SKCM cases with altered *RAB13* showed poor OS (both *P* < 0.05; Fig. 4D and F). However, the OV cases with altered *RAB13* showed better OS (*P* < 0.05; Fig. 4E). This shows that changes in the same gene play different roles in different tissues. Tumor mutational burden (TMB) was shown to be negatively connected with RAB13 in BRCA (*P* < 0.001), LGG (*P* < 0.01), PRAD (*P* < 0.001), PCPG (*P* < 0.05), and THYM (*P* < 0.001), but favorably correlated with RAB13 in SKCM (*P* < 0.01) (Fig. 4G). Additionally, RAB13 was negatively connected with mutant-allele tumor heterogeneity in COAD (*P* < 0.05), CESC (*P* < 0.05), and HNSC (*P* < 0.05), while it was favorably correlated with mutant-allele tumor heterogeneity in ACC (*P* < 0.05), ESCA (*P* < 0.01), and CHOL (*P* < 0.05). (Fig. 4H). The foregoing findings suggested that changed RAB13 warrants additional investigation.

### Genes closely associated with RAB13: a functional enrichment study

**Figure 5:**
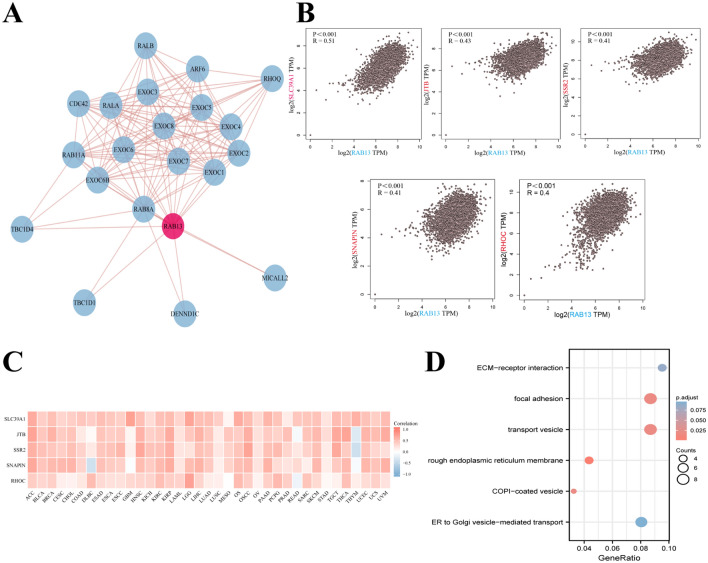
RAB13-related gene enrichment and pathway analysis. (**A**) Diagram of the interaction network between RAB13 and 20 Rab13-binding proteins. (**B**) RAB13 expression in TCGA cancers correlates with that of several representative genes (SLC39A1, JTB, SSR2, SNAPIN, and RHOC). (**C**) Expression heatmap showing relationships between RAB13 and SLC39A1, JTB, SSR2, SNAPIN, and RHOC. (**D**) GO|KEGG pathway analysis based on the RAB13-binding and interacted genes.

The STRING web tool further investigated the underlying molecular mechanism of the RAB13 in the emergence and evolution of cancers. Twenty proteins, including the PPI network, are shown to bind to RAB13 (Fig. 5A). Then, using GEPIA2, we compiled a list of the top 100 genes that were linked with RAB13 expression. The expression of RAB13 was found to be significantly related with SLC39A1, JTB, SSR2, SNAPIN, and RHOC (all *P* < 0.001; Fig. 5B). Next, we explored the association of these five genes with a variety of tumors and drew a heatmap (Fig. 5C). There was a clear positive correlation between RAB13 and the expression of these five genes across a wide range of malignancies. Moreover, we conducted KEGG and GO enrichment studies. According to the findings, these genes are most often linked to “focal adhesion” and “transport vesicle”, implying that these pathways in the impact of *RAB13* on the mechanism of tumorigenesis and development (Fig. 5D). The RAB family has been shown to play an important role in vesicle transport^4^, which is consistent with previous findings.

### Analysis of RAB13 immune infiltration data

**Figure 6:**
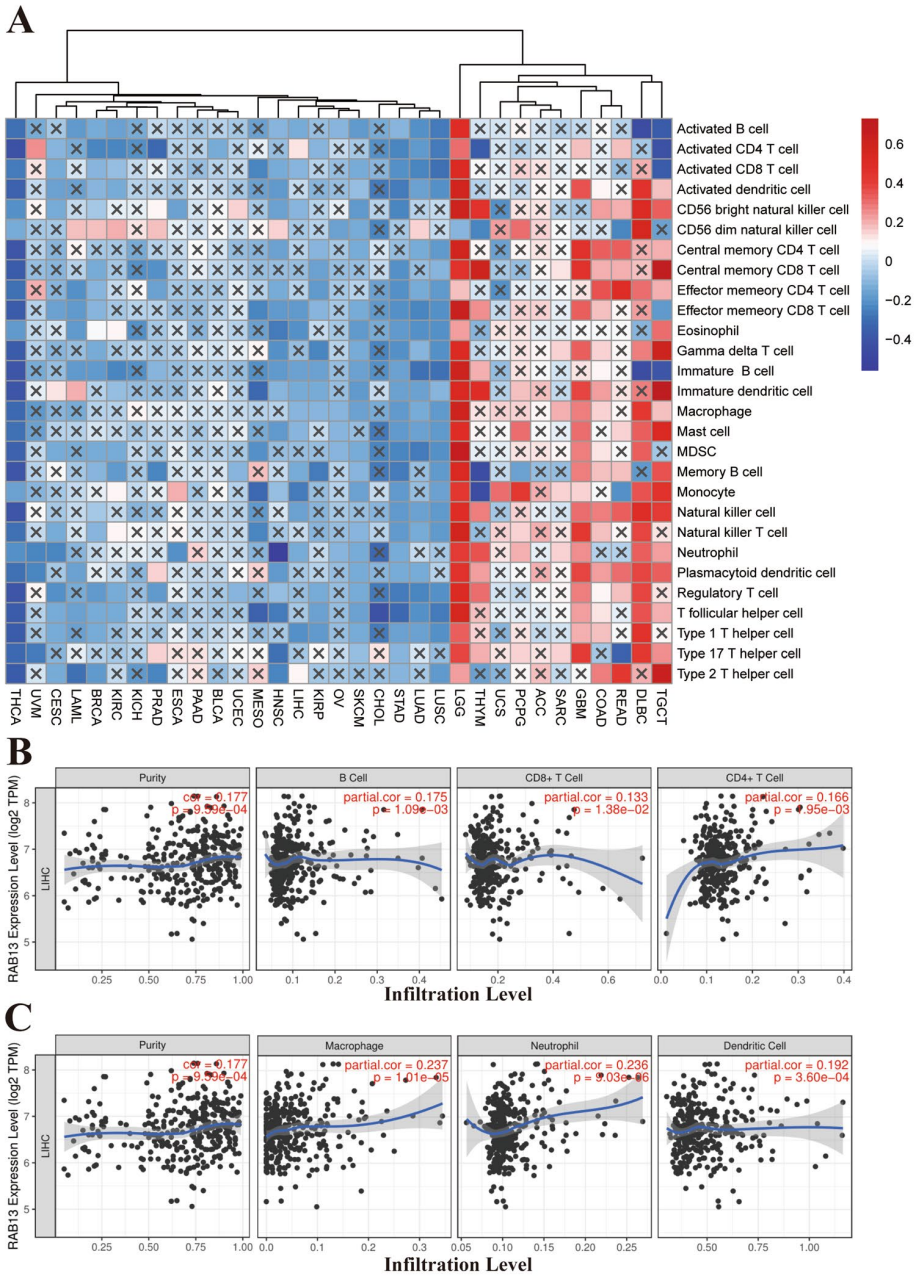
RAB13 immune infiltration study. (**A**) Effect of RAB13 on the infiltration of different immune cells in different tumors based on ssGESA. (**B**) Correlation between RAB13 expression and B cell, CD8 + T cell, CD4 + T cell in LIHC based on TIMER database. (**C**) Correlation between RAB13 expression and macrophage, neutrophil, dendritic cell in LIHC based on TIMER database.

Metabolic changes in tumor cells can affect immune cell function. Previous research has demonstrated that *RAB13* can affect immune responses^23,24^. That's why we employed ssGSEA to look at how RAB13 affects immune cell infiltration levels across different cancer types. From Fig. 6A, we can see that *RAB13* had different effects on immune cells in different tumors. Among them, *RAB13* had the most significant effect on LGG, which can up-regulate the expression of most immune cells. However, we zeroed particularly on RAB13 because of its possible impact on immunological infiltration into LIHC. We found that *RAB13* negatively regulated immune cell infiltration in LIHC (Fig. 6A). Immune cells play an immune role by secreting immune regulatory factors. Thus, we looked into how RAB13 expression is connected to immune regulatory variables. Similar to the previous results, *RAB13* can up-regulate or down-regulate the expression of immune regulatory factors. However, *RAB13* did not have obvious correlation with most immune factors in LIHC (Fig. S2). We then supplemented this with the TIMER database. There was a favorable correlation between RAB13 expression and the infiltration of B cell, CD8 + T cell, CD4 + T cell, macrophage, neutrophil and dendritic cell (all *P* < 0.05; Fig. 6B and C). The discrepancy with ssGSEA results may be due to different data sources.

### The RAB13-immune checkpoint-LIHC relationship

**Figure 7:**
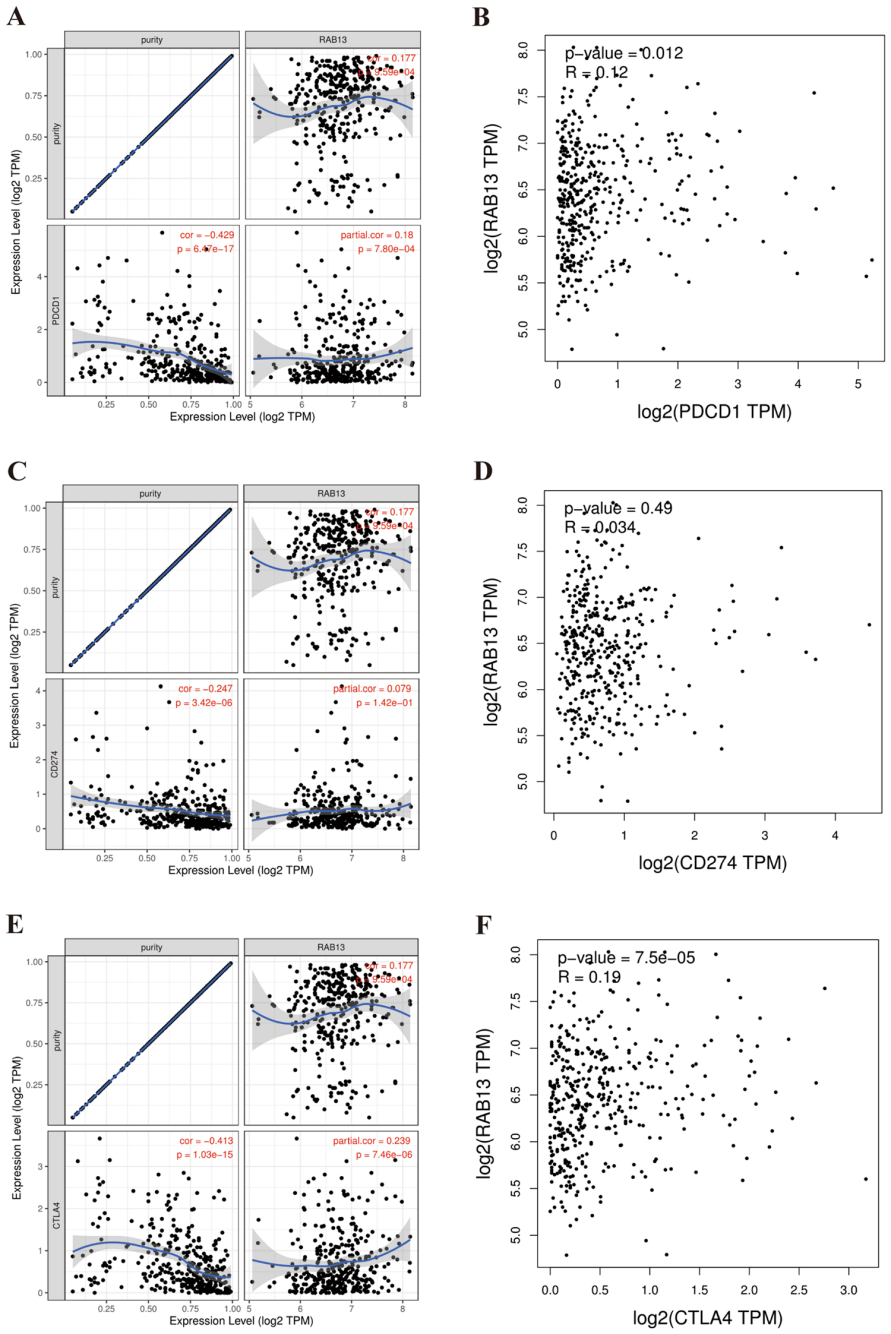
Expression of PD-1, PD-L1, and CTLA-4 are correlated with RAB13 expression in LIHC. Spearman correlation of RAB13 with expression of PD-1 (**A**), PDL1 (**C**), CTLA4 (**E**) in LIHC adjusted by purity using TIMER. The expression correlation of RAB13 with PD-1 (**B**), PDL1 (**D**), CTLA4 (**F**) in LIHC determined by GEPIA database.

PD1, PDL1 and CTLA4 are the most representative immune checkpoint pathways, which are closely related to tumor immune escape. Immunotherapy for LIHC has brought breakthroughs in the treatment of LIHC. Therefore, the correlation between *RAB13* and PD1, PDL1, and CTL4 was evaluated by TIMER database. After purity adjustment, we discovered a favorable correlation between RAB13 and the expression of PD1, PDL1, and CTLA4 in LIHC, (all *P* < 0.001, Fig. 7A,C,E). Then, we further verified the results using the GEPIA2 database. The results revealed that PD1 (*P* < 0.05) and CTLA4 (*P* < 0.001) were consistent with the previous results (Fig. 7B,F), however, PDL1 expression did not correlate with RAB13 expression. (Fig. 7D). These findings highlighted the significance of immune escape in RAB13-mediated carcinogenesis of LIHC.

### RAB13 participates in a number of LIHC biological processes

**Figure 8:**
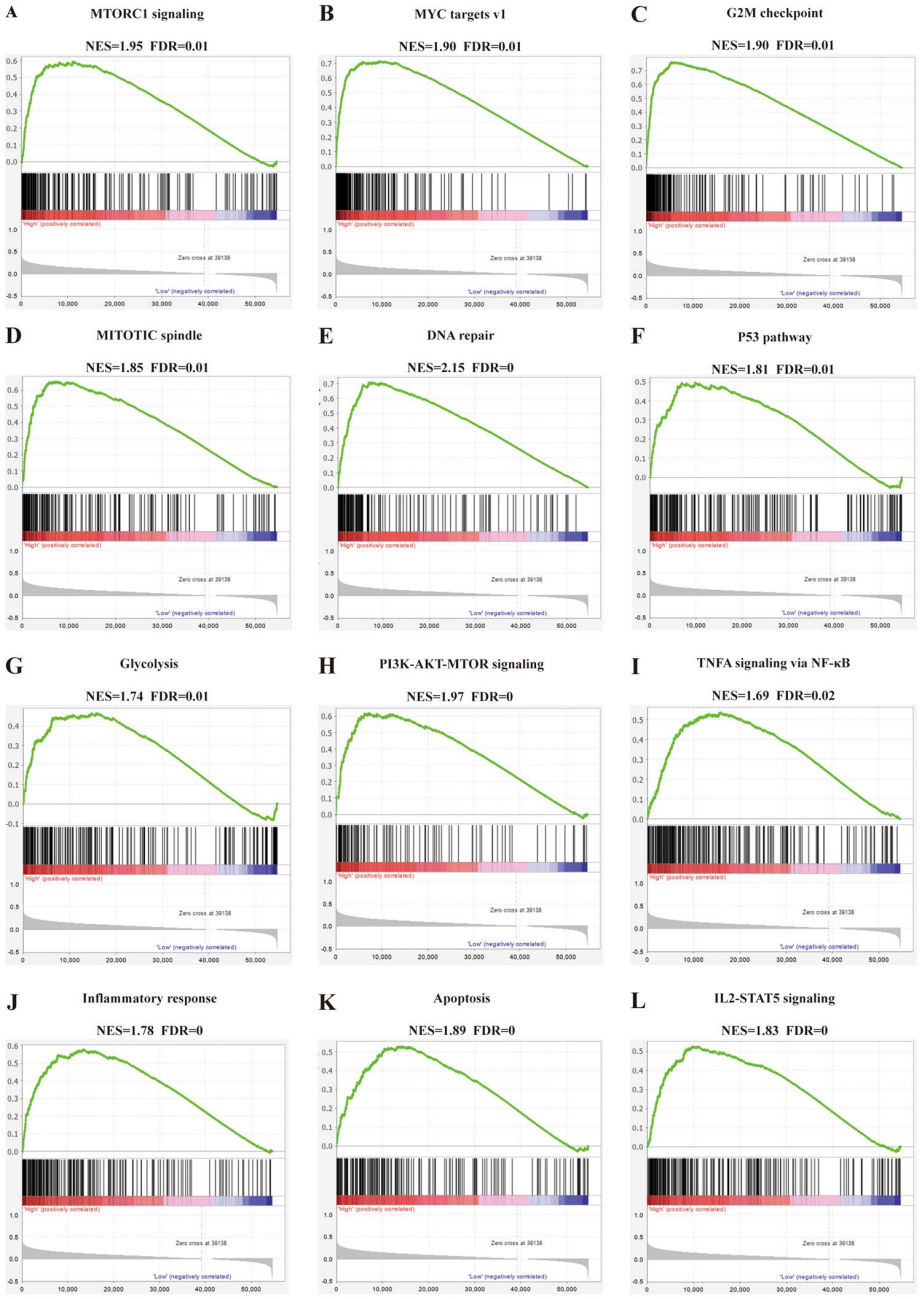
Gene Set Enrichment Analysis of RAB13 in LIHC. (**A**) MTORC1 signaling; (**B**) MYC targets v1; (**C**) G2M checkpoint; (**D**) MITOTIC spindle; (**E**) DNA repair; (**F**) P53 pathway; (**G**) Glycolysis; (**H**) PI3K-AKT-MTOR signaling; (**I**) NFA signaling via NF-κB; (**J**) Inflammatory response; (**K**) Apoptosis; (**L**) IL2-STAT5 signaling. *NES* normalized enrichment score, *FDR* false discovery rate.

According to the previous experimental results, we further explored the enrichment analysis of *RAB13* in LIHC by GSEA. According to the findings, six cell cycle-related pathways were considerably enriched, namely MTORC1 signaling, MYC targets v1, G2M checkpoint, MITOTIC spindle, DNA repair, and P53 pathway (Fig. 8A–F). High expression of *RAB13* can also promote LIHC metabolism through Glycolysis and PI3K-AKT-MTOR signaling (Fig. 8G,H). Additionally, the inflammatory response-related pathways, such as inflammatory response, and the immune response-related pathways, such as TNFA signaling via NF-B, were also enriched in LIHC (Fig. 8I,J). Finally, *RAB13* also facilitated the expression of apoptosis-related pathways in LIHC, such as Apoptosis and IL2-STAT5 signaling (Fig. 8K,L). These findings show a direct correlation between the progression of LIHC and high levels of RAB13 expression.

## Discussion

In the last few decades, the development of high-throughput technologies has upended biological and biomedical research. Researchers can comprehensively and systematically study the genome (genomics), the set of RNA molecules (transcriptomics) and the set of proteins of an organism^25,26^. With genomics, transcriptomics, protein sets exploding, methods for identifying tumor genes are developing rapidly. Databases are essential for identifying candidate disease genes and discriminating their function^27^. In addition, with the optimization of data preprocessing and algorithms, the accuracy and efficiency of biological information analysis are constantly improving^28,29^. The human genome has approximately 25,000 genes^30^. However, only a small number of genetic alterations cause tumors to develop and progress. Therefore, the study of bioinformatics technology in identifying tumors is crucial. In this study, we utilized bioinformation analysis technology and numerous databases to thoroughly analyze the function of RAB13 in cancers. RAB13 was shown to be significantly expressed in tumor tissues, which indicated a poor prognosis. This is consistent with the conclusion in Fig. 4A that RAB13 is the most frequently occurring mutation in most tumors. We did observe, nonetheless, that RAB13 expression in LAML tumor tissues was lower than it was in the nearby tumor tissues. This may be due to the different mutation types of RAB13 in LAML.

The nucleotide sequence of an organism, virus, or extrachromosomal DNA genome changes when there is a mutation. Human genome mutations come in many different forms. The diversity of gene mutations and susceptibility to cancer have always been the focus of research. Here, we evaluated the major types of RAB13 mutations in tumors. We discovered that amplification was the most prevalent form of mutation in RAB13. Amplification refers to a rise in the number of copies of a certain DNA sequence or gene in a cell's genome, which can result in the overexpression of a protein or the activation of an oncogene. This is consistent with the conclusion in Fig. 1A. Amplification can lead to activation of oncogenes and inactivation of tumor suppressor genes and resistance to chemotherapy^31^. Interestingly, we discovered that RAB13 mutations were associated with a poor prognosis in ACC and SKCM, but a prolonged survival rate in OV. This may be due to tissue-specific differences in gene expression and function. The amounts of mutations in a tumor is measured by the tumor mutational burden, and it may be a good predictive biomarker^32^. Mutant-allele tumor heterogeneity is an index for evaluating genetic heterogeneity within tumors. For the detection and management of malignancies, these two indications are quite helpful. Here, we also evaluated TMB and mutant-allele tumor heterogeneity.

To explore the mechanism of *RAB13* affecting tumorigenesis and development, we identified genes co-expressed with *RAB13*. According to the outcomes, the expression of SLC39A1, JTB, SSR2, SNAPIN, RHOC and RAB13 was strongly correlated. And the enrichment analysis showed that the transport vesicle was significantly enriched in the appeal genes. Transport vesicles are membrane-bound structures that transport chemicals and other cellular components between compartments and between cells. They are involved in various cellular processes such as protein secretion, intracellular transport, and intercellular communication. etc.^4^. Studies have confirmed that JBT^33^, RHOC^34^, and SNAPN^35^ play an important role in vesicle transport. However, no studies have demonstrated the association of SLC39A1 and SSR2 with RAB13. RAB13 promotes the occurrence and growth of tumors and is highly correlated with appeal genes. Our research provides clues to the correlation between RAB13 and appeal genes, providing new therapeutic targets for tumor treatment. However, more research is still needed to determine the precise regulatory mechanisms that control RAB13 and the above genes and clarify the role of SLC39A1 and SSR2 in vesicle transport.

A tumor's ability to initiate, develop, metastasize, and respond to therapy are all influenced by its microenvironment (TME). Immune cells, stromal cells, blood arteries, and extracellular matrix are all components of the dynamic TME. In principle, protection mechanisms in the body's immune system acts as the body's guard, which can divide into cytotoxic innate and adaptive immunity, suppressing the development and progression of tumors. But it turned out that immune cells actually play a dual role in tumors. It has been found that macrophages promote the development and malignant progression of cancer^36^. Colorectal cancer risk is raised by immune dysregulation in inflammatory bowel disease^37^. The response of tumors to radiation and chemotherapy is also greatly influenced by immune cell infiltration, as has been established in a large number of studies^38,39^. Our experimental data show that the infiltration of B cell, CD8 + T cell, CD4 + T cell, macrophage, neutrophil, dendritic cell and *RAB13* was positively related in LIHC. This provides a solid foundation for future therapies targeting immune cell infiltration in LIHC.

Receptors for the molecule that triggers cell death and their ligands make up the immune system's checkpoints. Improving cancer care dramatically, immune checkpoint inhibitors (ICIs) have recently been developed. Immunotherapy has been successfully applied to the treatment of numerous malignancies, including lung cancer^40^, hepatocellular carcinoma^41^, gastric cancer^42^, breast cancer^43^ and others. At present, PDCD1, CD274 and CTLA4 are the most widely used immune checkpoints in LIHC^44^, and immunotherapy drugs targeting them have greatly improved the prognosis of LIHC. Therefore, we looked for a connection between RAB13 expression and these markers using LIHC and found a positive one between RAB13 and PDCD1, CD274, and CTLA4. Hence, measuring RAB13 expression can be used as a proxy for the sensitivity to various immune checkpoint inhibitors. In the future, to improve the therapeutic effect of tumors, more immune checkpoints need to be found.

In brief, our study preliminarily revealed the role of *RAB13* in pan-cancers. We suggest that further explorations of *RAB13*-related experiments should be warranted to dig its specific functions in tumors, which may bring new strategies and targets for tumor treatment. We do have some caveats in our study, though. Despite the fact that we have combined data from numerous sources, more in vivo and in vitro experiments are required to confirm our main findings and translate their clinical value.

## Conclusion

In this study, we looked at the effects of RAB13 on pan-carcinoma from every angle possible, including its impact on survival, prognosis, genetic and epigenetic alterations. Based on our findings, RAB13 appears to have a significant impact on both LIHC and LGG. Among them, we focused on LIHC. RAB13 has been shown to participate in a number of cellular functions and signaling pathways of LIHC, and it can also affect the sensitivity of LIHC to immunotherapy. This research adds important knowledge to the understanding and treatment of cancers, particularly LIHC.

### Supplementary Information

Supplementary Figures.

## Data Availability

The datasets analysed during the current study are available in the TIMER2.0 (http://timer.cistrome.org/), GEPIA 2 (http://gepia2.cancer-pku.cn/#index), UALCAN (http://ualcan.path.uab.edu/analysis-prot.html), cBioPortal (https://www.cbioportal.org/), TIMER (https://cistrome.shinyapps.io/timer/), STRING https://cn.string-db.org/) database. Contact the corresponding Author for additional resources.

## Abbreviations

RAB: Ras-related in brain
OS: Overall survival
DFS: Disease-free survival
sEVs: Small extracellular vesicles
TIMER: Tumor immune estimation resource
GEPIA: Gene expression profiling interactive analysis
UALCAN: The University of aLabama at Birmingham CANcer data analysis portal
CPTAC: Clinical proteomic tumor analysis consortium
ssGSEA: Single sample gene set enrichment analysis
GSEA: Gene set enrichment analysis
PPI: Protein–protein interaction
GO: Gene ontology
KEGG: Kyoto encyclopedia of genes and genomes
TMB: Tumor mutational burden
TME: Tumor microenvironment
ICIs: Immune checkpoint inhibitors

## References

[1] Chen F, Wendl MC, Wyczalkowski MA, Bailey MH, Li Y, Ding L (2021). Moving pan-cancer studies from basic research toward the clinic. Nat. Cancer.

[2] Sung H, Ferlay J, Siegel RL, Laversanne M, Soerjomataram I, Jemal A (2021). Global cancer statistics 2020: GLOBOCAN estimates of incidence and mortality worldwide for 36 cancers in 185 countries. CA Cancer J. Clin..

[3] Kang MJ, Won YJ, Lee JJ, Jung KW, Kim HJ, Kong HJ (2022). Cancer statistics in Korea: Incidence, mortality, survival, and prevalence in 2019. Cancer Res. Treat..

[4] Zhen Y, Stenmark H (2015). Cellular functions of Rab GTPases at a glance. J. Cell Sci..

[5] Cho SH, Kuo IY, Lu PF, Tzeng HT, Lai WW, Su WC (2018). Rab37 mediates exocytosis of secreted frizzled-related protein 1 to inhibit Wnt signaling and thus suppress lung cancer stemness. Cell Death Dis..

[6] Yousaf M, Ali M (2020). Modulation of ABCG2 surface expression by Rab5 and Rab21 to overcome multidrug resistance in cancer cells. Xenobiotica.

[7] McBrayer SK, Cheng JC, Singhal S, Krett NL, Rosen ST, Shanmugam M (2012). Multiple myeloma exhibits novel dependence on GLUT4, GLUT8, and GLUT11: Implications for glucose transporter-directed therapy. Blood.

[8] Sun Y, Bilan PJ, Liu Z, Klip A (2010). Rab8A and Rab13 are activated by insulin and regulate GLUT4 translocation in muscle cells. Proc. Natl. Acad. Sci. U. S. A..

[9] Hinger SA, Abner JJ, Franklin JL, Jeppesen DK, Coffey RJ, Patton JG (2020). Rab13 regulates sEV secretion in mutant KRAS colorectal cancer cells. Sci. Rep..

[10] Sahgal P, Alanko J, Icha J, Paatero I, Hamidi H, Arjonen A (2019). GGA2 and RAB13 promote activity-dependent beta1-integrin recycling. J. Cell Sci..

[11] Wang H, Xu H, Chen W, Cheng M, Zou L, Yang Q (2022). Rab13 sustains breast cancer stem cells by supporting tumor-stroma cross-talk. Cancer Res..

[12] Ioannou MS, Bell ES, Girard M, Chaineau M, Hamlin JN, Daubaras M (2015). DENND2B activates Rab13 at the leading edge of migrating cells and promotes metastatic behavior. J. Cell Biol..

[13] Chen P, Chen G, Wang C, Mao C (2019). RAB13 as a novel prognosis marker promotes proliferation and chemotherapeutic resistance in gastric cancer. Biochem. Biophys. Res. Commun..

[14] Li T, Fu J, Zeng Z, Cohen D, Li J, Chen Q (2020). TIMER2.0 for analysis of tumor-infiltrating immune cells. Nucleic Acids Res..

[15] Tang Z, Kang B, Li C, Chen T, Zhang Z (2019). GEPIA2: An enhanced web server for large-scale expression profiling and interactive analysis. Nucleic Acids Res..

[16] Chandrashekar DS, Karthikeyan SK, Korla PK, Patel H, Shovon AR, Athar M (2022). UALCAN: An update to the integrated cancer data analysis platform. Neoplasia.

[17] Gao J, Aksoy BA, Dogrusoz U, Dresdner G, Gross B, Sumer SO (2013). Integrative analysis of complex cancer genomics and clinical profiles using the cBioPortal. Sci. Signal..

[18] Hanzelmann S, Castelo R, Guinney J (2013). GSVA: Gene set variation analysis for microarray and RNA-seq data. BMC Bioinform..

[19] Szklarczyk D, Gable AL, Nastou KC, Lyon D, Kirsch R, Pyysalo S (2021). The STRING database in 2021: customizable protein-protein networks, and functional characterization of user-uploaded gene/measurement sets. Nucleic Acids Res..

[20] Tilford CA, Siemers NO (2009). Gene set enrichment analysis. Methods Mol. Biol..

[21] Srikanth K, Lee SH, Chung KY, Park JE, Jang GW, Park MR (2020). A gene-set enrichment and protein-protein interaction network-based GWAS with regulatory SNPs identifies candidate genes and pathways associated with carcass traits in Hanwoo cattle. Genes (Basel).

[22] Yu G, He QY (2016). ReactomePA: An R/Bioconductor package for reactome pathway analysis and visualization. Mol. Biosyst..

[23] Lu Y, Chan YT, Tan HY, Zhang C, Guo W, Xu Y (2022). Epigenetic regulation of ferroptosis via ETS1/miR-23a-3p/ACSL4 axis mediates sorafenib resistance in human hepatocellular carcinoma. J. Exp. Clin. Cancer Res..

[24] Zhang ZD, Li HX, Gan H, Tang Z, Guo YY, Yao SQ (2022). RNF115 inhibits the post-ER trafficking of TLRs and TLRs-mediated immune responses by catalyzing K11-linked ubiquitination of RAB1A and RAB13. Adv. Sci. (Weinh).

[25] Velculescu VE, Zhang L, Zhou W, Vogelstein J, Basrai MA, Bassett DE (1997). Characterization of the yeast transcriptome. Cell.

[26] Anderson NL, Anderson NG (1998). Proteome and proteomics: New technologies, new concepts, and new words. Electrophoresis.

[27] van Driel MA, Brunner HG (2006). Bioinformatics methods for identifying candidate disease genes. Hum. Genom..

[28] Ghareyazi A, Kazemi A, Hamidieh K, Dashti H, Tahaei MS, Rabiee HR (2022). Pan-cancer integrative analysis of whole-genome De novo somatic point mutations reveals 17 cancer types. BMC Bioinform..

[29] Srivatsa S, Montazeri H, Bianco G, Coto-Llerena M, Marinucci M, Ng CKY (2022). Discovery of synthetic lethal interactions from large-scale pan-cancer perturbation screens. Nat. Commun..

[30] Gonzaga-Jauregui C, Lupski JR, Gibbs RA (2012). Human genome sequencing in health and disease. Annu. Rev. Med..

[31] Fischer U, Meese E (2022). Gene amplification in tumor cells: Developed de novo or adopted from stem cells. Cells.

[32] Büttner R, Longshore JW, López-Ríos F, Merkelbach-Bruse S, Normanno N, Rouleau E (2019). Implementing TMB measurement in clinical practice: Considerations on assay requirements. ESMO Open.

[33] Jayathirtha M, Neagu AN, Whitham D, Alwine S, Darie CC (2022). Investigation of the effects of overexpression of jumping translocation breakpoint (JTB) protein in MCF7 cells for potential use as a biomarker in breast cancer. Am. J. Cancer Res..

[34] Eckenstaler R, Hauke M, Benndorf RA (2022). A current overview of RhoA, RhoB, and RhoC functions in vascular biology and pathology. Biochem. Pharmacol..

[35] Ghildiyal R, Gabrani R (2021). Deciphering the human cellular interactors of alphavirus unique domain of chikungunya virus. Virus Res..

[36] Qian BZ, Pollard JW (2010). Macrophage diversity enhances tumor progression and metastasis. Cell.

[37] Lakatos PL, Lakatos L (2008). Risk for colorectal cancer in ulcerative colitis: Changes, causes and management strategies. World J. Gastroenterol..

[38] Waniczek D, Lorenc Z, Snietura M, Wesecki M, Kopec A, Muc-Wierzgon M (2017). Tumor-associated macrophages and regulatory T cells infiltration and the clinical outcome in colorectal cancer. Arch. Immunol. Ther. Exp. (Warsz).

[39] Lyu L, Yao J, Wang M, Zheng Y, Xu P, Wang S (2020). Overexpressed pseudogene HLA-DPB2 promotes tumor immune infiltrates by regulating HLA-DPB1 and indicates a better prognosis in breast cancer. Front. Oncol..

[40] Steven A, Fisher SA, Robinson BW (2016). Immunotherapy for lung cancer. Respirology.

[41] Sangro B, Sarobe P, Hervás-Stubbs S, Melero I (2021). Advances in immunotherapy for hepatocellular carcinoma. Nat. Rev. Gastroenterol. Hepatol..

[42] Kelly RJ (2017). Immunotherapy for esophageal and gastric cancer. Am. Soc. Clin. Oncol. Educ. Book.

[43] Emens LA (2018). Breast cancer immunotherapy: Facts and hopes. Clin. Cancer Res..

[44] Wang J, Li J, Tang G, Tian Y, Su S, Li Y (2021). Clinical outcomes and influencing factors of PD-1/PD-L1 in hepatocellular carcinoma. Oncol. Lett..

