# Supplementary material for: Clinical implications of RAB13 expression in pan-cancer based on multi-databases integrative analysis
Source: Sci Rep. 2023 Oct 6;13:16859. doi: 10.1038/s41598-023-43699-2 (PMC10558570; doi:10.1038/s41598-023-43699-2)
Supplement: Supplementary file 1 — Supplementary Figures. [file 41598_2023_43699_MOESM1_ESM.docx]

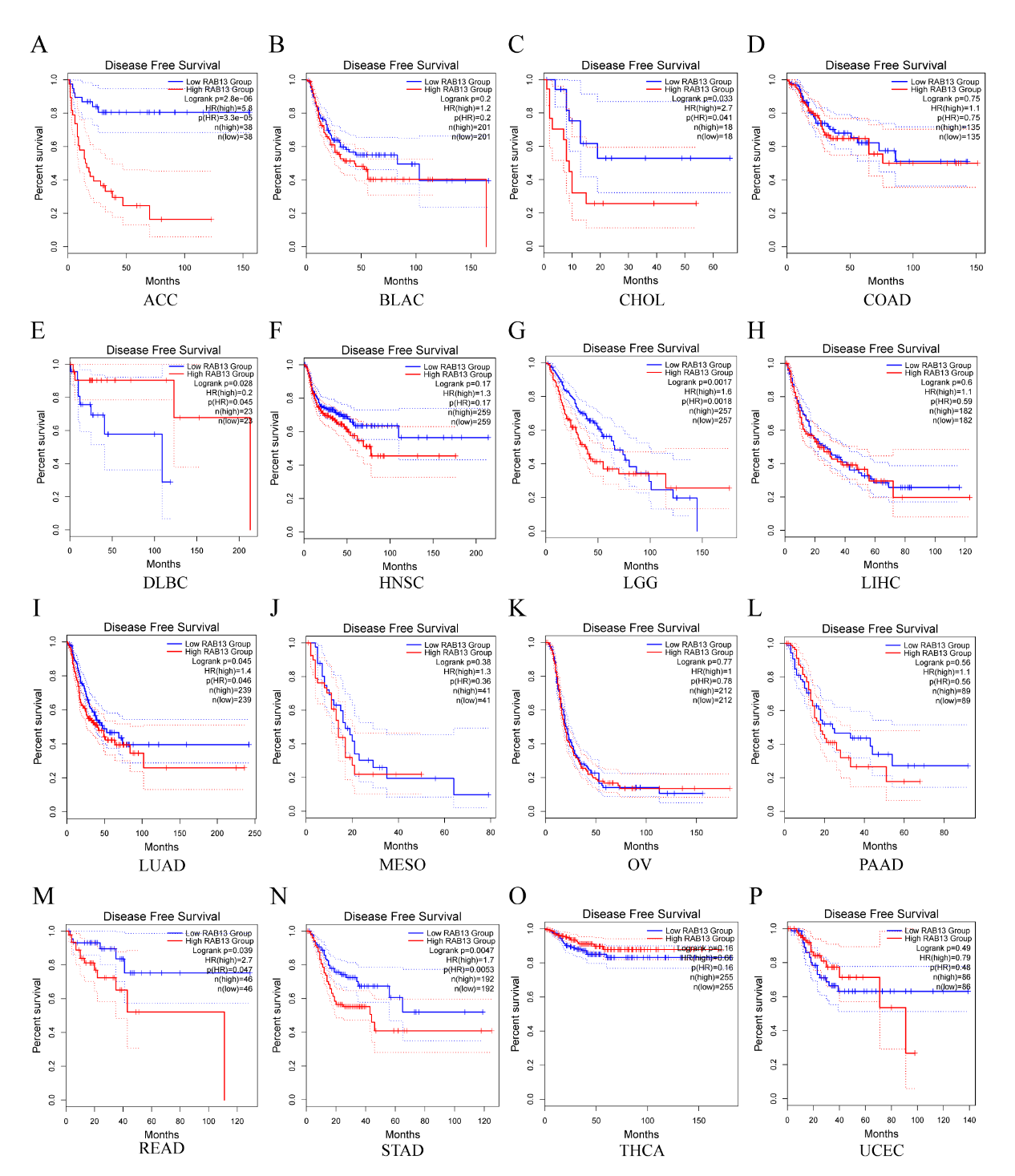


**Figure S1.** Based on data from the GEPIA 2 database, the relationship between RAB13 expression and DFS in various malignancies. (**A**)ACC, (**B**)BLAC, (**C**)CHOL, (**D**)COAD, (**E**)DLBC, (**F**)HNSC, (**G**)LGG, (**H**)LIHC, (**I**)LUAD, (**J**)MESO, (**K**)OV, (**L**)PAAD, (**M**)READ, (**N**)STAD, (**O**)THCA, (**P**)UCEC.


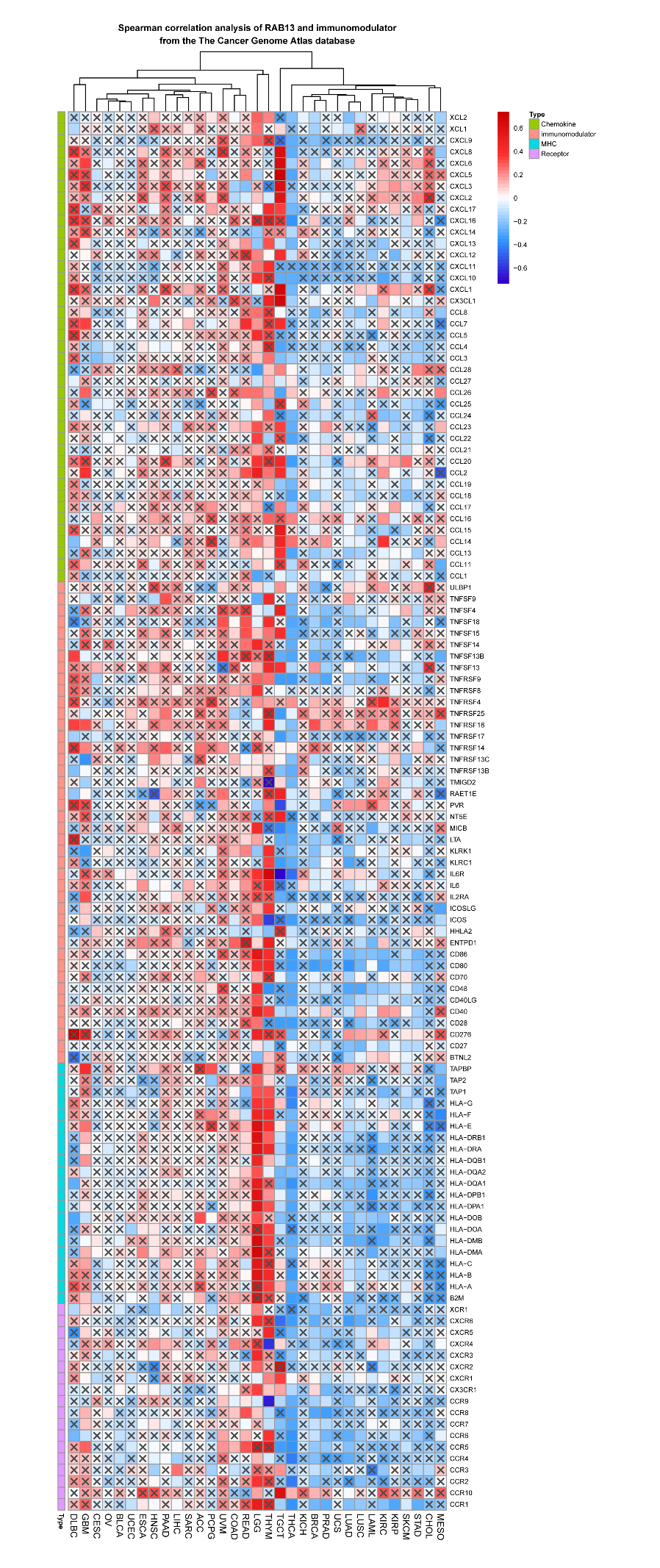


**Figure S2.** RAB13 and ssGESA-based immunomodulator correlation analyses using the Spearman method.
